# Knockout of OR39 reveals redundancy in the olfactory pathway regulating the acquisition of host seeking in *Anopheles coluzzii*

**DOI:** 10.1098/rspb.2023.2092

**Published:** 2023-11-29

**Authors:** Annika Hinze, Julien Pelletier, Majid Ghaninia, Eric Marois, Sharon Rose Hill, Rickard Ignell

**Affiliations:** ^1^ Disease Vector Group, Unit of Chemical Ecology, Department of Plant Protection Biology, Swedish University of Agricultural Sciences, Alnarp 750 07, Sweden; ^2^ School of Life Sciences, Arizona State University, Tempe, AZ, USA; ^3^ Inserm, CNRS, Université de Strasbourg, Strasbourg 67070, France

**Keywords:** CRISPR–Cas9, human odour, mosquito flight, odour valence, SSR, wind tunnel

## Abstract

The attraction of anthropophilic mosquitoes to human host cues, such as body odour and carbon dioxide, gradually increases during adult maturation. This acquisition of host-seeking behaviour correlates with age-dependent changes in odorant receptor (OR) transcript abundance and sensitivity of olfactory sensory neurons (OSNs). One OR gene of the human malaria vector, *Anopheles coluzzii*, *AcolOR39*, is significantly downregulated in mature females, and a cognate ligand of AcolOR39, sulcatone, a major component of human emanations, mediates the observed behavioural inhibition of newly emerged (teneral) females to human body odour. Knockout of *AcolOR39*, using CRISPR–Cas9 mutagenesis, selectively abolished sulcatone detection in OSNs, housed in trichoid sensilla. However, knockout of *AcolOR39* altered neither the response rate nor the flight behaviour of teneral females in a wind tunnel, indicating the involvement of other genes, and thus a redundancy, in regulating the acquisition of host seeking in mosquitoes.

## 1. Background

For a host-seeking mosquito, approaching a potential host to obtain a blood meal for egg production involves a substantial risk of being killed by host-defensive behaviour [1,2], such as swatting in many mammals [3] or foot-pecking in birds [1]. Even host-defensive behaviours with a less fatal outcome, resulting in a lower feeding success rate, decrease the survival and reproduction of the mosquito [1,4]. Thus, it is a crucial determinant of female fitness to host seek exclusively when females are in the correct physiological state, which includes the maturity of the adult, among other endogenous factors [5–7]. Newly emerged (teneral) females are not able to take a blood meal since their mouthparts and other organs are not yet fully developed, and they do not respond to, or even avoid, human odour [5,8–12]. Within the first few days after adult emergence, female mosquitoes undergo physiological and behavioural changes that make them competent to host seek and take a blood meal [5]. During this period, the response to host-associated cues, such as carbon dioxide (CO_2_) and volatile organic compounds (VOCs) emitted from breath and skin, gradually increases, accompanied by a gain in the sensitivity of olfactory sensory neurons (OSNs) in the antennae and maxillary palps, tuned to salient host VOCs and CO_2_ [10,11,13,14]. Several studies have demonstrated a strong correlation between female age, onset of host seeking, changes in the sensitivity of the peripheral olfactory system and the differential expression of chemosensory genes, such as odorant binding proteins, odorant receptors (ORs), as well as gustatory and ionotropic receptors [10–12,14]. While it is an accepted hypothesis that the behavioural acquisition of host seeking correlates with changes in the expression level of certain chemosensory genes [10–12,14], the causative molecular mechanism remains unclear.

Omondi *et al*. [11] investigated the onset of host seeking in one of the main vectors of malaria, *Anopheles coluzzii*, and identified several candidate *OR*s, which encode receptors tuned to VOCs present in human odour, whose age-dependent change in expression level was associated with an increase in both behavioural attraction and physiological sensitivity to human odour. Removing the main ligands of one of these receptors, AcolOR39, from a synthetic blend mimicking human odour, did not change attraction of mature females in a two-choice assay. However, when these two VOCs, 1-hexanol and sulcatone, were tested on their own, mature females still preferred the two-compound blend, while teneral females preferred the control, replicating the reported age-dependent shift in attraction, and thus suggesting that these compounds regulate the age-dependent differential attractiveness to human odour. While 1-hexanol and sulcatone are not uniquely detected by AcolOR39 [11,15], it is the only OR sensitive to these compounds to exhibit an age-dependent downregulation, indicating a potential role in the acquisition of host seeking in *A. coluzzii* [11].

The present study investigated the role of AcolOR39 (hereafter OR39), by CRISPR–Cas9 knockout, in *A. coluzzii* (previously *A. gambiae*, molecular M form; [16]) in the detection of human-associated VOCs, using single sensillum recordings (SSRs), and the acquisition of host seeking, by high-resolution flight assays in a wind tunnel. Since the expression of *OR39* is downregulated during adult maturation, we hypothesize that OR39 acts within a pathway signalling behavioural inhibition to a salient host VOC (i.e. sulcatone) in teneral females (see also [11]). We test the hypothesis that the decrease in *OR39* abundance during adult maturation nullifies the inhibitory signal in mature females competent for host seeking.

## 2. Methods

### (a) Mosquito rearing

*Anopheles coluzzii* (wild-type: G3 strain; lines for mutagenesis, transgenic lines: see below) were reared and maintained at 27 ± 1°C and 65 ± 5% relative humidity under a 12 h light:12 h dark regimen. Eggs were hatched in larval trays (24 cm × 18 cm × 7.5 cm), filled with approximately 2 cm of distilled water, and emerging larvae fed on Tetramin Baby fish food (Tetra, Melle, Germany). Pupae were transferred to Bugdorm cages (17.5 cm × 17.5 cm × 17.5 cm; MegaView Science, Taichung City, Taiwan), in which adult mosquitoes were provided *ad libitum* access to a 10% sucrose solution. For propagation of the colonies, adult females were fed on donor sheep blood (Håtunalab, Bro, Sweden) using a membrane feeding system (Hemotek, Blackburn, UK), and provided with wet filter papers for oviposition. Mosquitoes 1-day post-emergence (dpe) were considered as teneral, whereas 4 dpe were considered as mature.

### (b) *OR39* knockout-lines

#### (i) Mutagenesis of OR39 knockout-lines

Site-directed mutagenesis of *OR39* (AGAP002639) was performed using the CRISPR–Cas9 system [17]. Genomic DNA was extracted from *A. coluzzii* (DNeasy Blood and Tissue Kit; Qiagen, Hilden, Germany) and used as template for amplifying a 1036 base-pair (bp) fragment, including the entire 5′ end of the gene, using PCR (Gotaq G2 DNA polymerase; Promega, Madison, USA) with the following primers: *OR39*f: 5′-ATGGTGTCGTTTGGTGCT-3′ and *OR39*r: 5′-TAGACGAGCAGCACATTCGG-3′; and programme: initial denaturation at 95°C for 2 min, 34 cycles at 95°C for 20 s, 58°C for 20 s and 72°C for 1 min followed by a final extension at 72°C for 5 min. The PCR product was purified (QIAquick Gel Extraction Kit; Qiagen) and sequenced (Eurofins Genomics, Ebersberg, Germany) in order to manually identify a suitable protospacer for guide RNA (gRNA) design. The protospacer (5′-GGCAGCGATGCACTCTTCAG-3′) was selected to include a native EarI (Eam1104I) recognition site (underlined), overlapping with the expected location of the CRISPR-mediated double-strand DNA break, for genotyping purposes. The protospacer was inserted into a pDSA-U6 transgenesis plasmid (electronic supplementary material, file S1) downstream of a U6 promoter, and upstream of the CRISPR-tracer scaffold [18], to generate a gRNA expression construct. Briefly, the plasmid was linearized by BbsI digestion (New England Biolabs, Hitchin, UK) and purified (QIAquick Gel Extraction Kit; Qiagen). The following primers containing sticky ends (underlined), complementary with BbsI overhangs on the digested plasmid, *OR39*-gRNAf: 5′-CCTTGGCAGCGATGCACTCTTCAG-3′ and *OR39*-gRNAr: 5′-AAACCTGAAGAGTGCATCGCTGCC-3′, were annealed by combining 5 µl of each primer (at 100 µM) in 40 µl nuclease-free water, incubating at 95°C for 5 min and cooling to room temperature (RT) over 1 h, and then diluted to 0.03 µM before ligation. The ligation reaction included 1 µl of annealed primers, 10 µl of BbsI-digested plasmid (equivalent to 20 ng), 4 µl of 5× T4 DNA ligase buffer, 0.2 µl of T4 DNA ligase (Invitrogen, Thermo Fisher Scientific, Waltham, USA) and 4.8 µl nuclease-free water in a final volume of 20 µl. The reaction was incubated for 1 h at 25°C, and 1 µl was used to transform OneShot TOP10 competent cells (Invitrogen). Positive clones were selected on Luria broth plates supplemented with 30 g ml^−1^ kanamycin, cultured, purified (QIAprep Spin Miniprep Kit; Qiagen) and sequenced (Eurofins Genomics) to confirm the integrity of the construct. The verified pDSA-U6-*OR39*gRNA plasmid was cultured, purified (Plasmid Midi Kit; Qiagen) and injected into *A. coluzzii* embryos of docking line X1 [19]. Resulting adults were crossed to wild-type mosquitoes and their progeny screened for fluorescence to establish a transgenic line. The mutation was induced by crossing transgenic insects of the *OR39* gRNA-expressing line with insects from a previously established Cas9-expressing transgenic line to produce F_1_ mosquitoes, combining both gRNA and Cas9 expression in their germline cells. The F_1_ males were then crossed with wild-type females to produce F_2_ insects heterozygous for potential mutations at the target locus.

#### (ii) Establishment of stable OR39 mutant lines

Mosquito genotyping was performed using the Phire Tissue Direct PCR Master Mix (Thermo Fisher Scientific) protocol to identify potential mutations at the expected locus in live insects. Genomic DNA was extracted from individual cold-anesthetized mosquitoes by dissecting one hind leg into 20 µl of extraction buffer and 0.5 µl of DNA Release Additive (Thermo Fisher Scientific). Each sample was incubated for 5 min at RT, and then 2 min at 98°C. The PCR amplification of a 648 bp fragment, spanning the mutagenesis target site, was performed in a final volume of 15 µl, including 1 µl of genomic DNA and 0.5 µl of each primer (10 µM; *OR39*f2: 5′-TGCAACTGGAGCAACAGATT-3′; *OR39*r: 5′-TAGACGAGCAGCACATTCGG-3′), using the following programme: initial denaturation at 98°C for 5 min, 34 cycles at 98°C for 5 s, 62°C for 5 s and 72°C for 20 s and a final extension at 72°C for 1 min. The PCR products were digested with FastDigest EarI (Eam1104I; Thermo Fisher Scientific), by adding 10 µl of digestion mix (including 0.3 µl of enzyme, 1 µl of FastDigest buffer and 8.7 µl nuclease-free water) directly into individual PCR products, and incubating for 3 h at 37°C. Digestion products were loaded onto a 1.5% agarose gel for electrophoresis at 85 V for 40 min and then visualized using GelRed Nucleic Acid Gel Stain (10 µl; Biotium, Fremont, USA) under UV light. Differences in migration profiles were used to identify mutant insects, with mutant alleles being protected from EarI digestion, and remaining uncut, whereas wild-type alleles were fully digested. Multiple candidate mutant F2 females were mated with wild-type males, and allowed to oviposit. The genomic DNA of these founder females was extracted (DNeasy Blood and Tissue Kit; Qiagen) and amplified using the primers *OR39*f2–*OR39*r (Gotaq G2 DNA polymerase; Promega), and the PCR products purified (QIAquick Gel Extraction Kit; Qiagen) and sequenced (Eurofins Genomics) to confirm the mutation. The progeny of two founder females with independent five-nucleotide deletion events were used to establish two stable homozygous *OR39* knockout mutant lines (*OR39*^42/42^ and *OR39*^117/117^) through five backcrosses with wild-type mosquitoes and a final selection of heterozygous, and then homozygous insects. The genotyping protocol described above was used to identify mutant insects throughout the different crossing stages, as well as for regular genotyping of the mutant lines to ensure homozygosity.

### (c) Flight behaviour

#### (i) The wind tunnel setup, video capture and odour presentation

**Figure 1.**
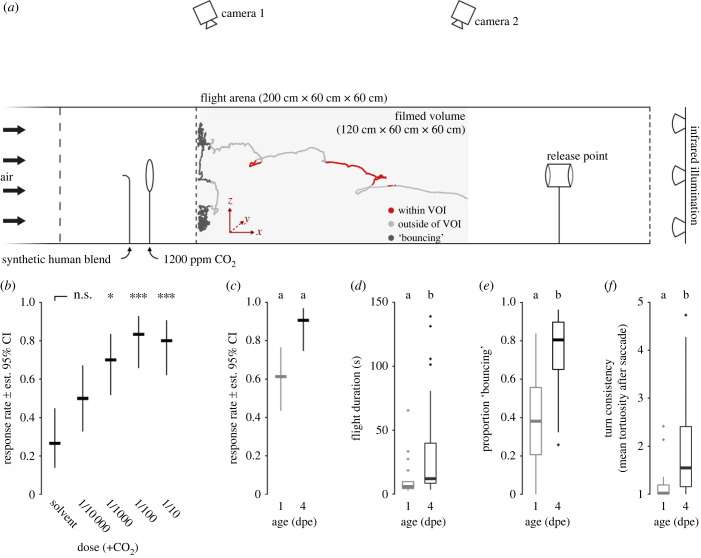
Mosquito flight and the acquisition of host seeking. (*a*) Schematic of the flight arena, odour delivery and video capture system. A representative flight trajectory of a mature (4-days post-emergence; dpe) wild-type *Anopheles coluzzii* in response to the optimal dose of the synthetic human blend and 1200 ppm carbon dioxide (CO_2_) is given within the filmed volume (covered by both cameras; grey box). VOI, volume of interest. (*b*) Response rate of mature wild-type mosquitoes to increasing doses of the synthetic human blend and CO_2_ (experiment I). Bars denote the estimated 95% confidence interval and asterisks significant differences for pairwise comparisons to the solvent, using ‘emmeans’ corrected for multiple comparisons with the Tukey method. *n*(per dose) = 30, *p* < 0.05. (*c*) Response rate of teneral (1 dpe) and mature (4 dpe) wild-type females to the synthetic human blend and CO_2_ (experiment II). Different letters denote significant differences between the genotypes (pairwise comparison using ‘emmeans’, corrected with the Tukey method). *n*(per treatment) = 31–32, *p* < 0.05. (*d*) Flight duration, (*e*) proportion of flight spent ‘bouncing’ at the upwind screen and (*f*) turn consistency (i.e. the mean tortuosity after a saccade). Boxes represent upper and lower quartiles, whiskers denote 1.5 times interquartile distance, crosses outliers and thick horizontal lines the median. Different letters denote significant differences between the treatments (Dunn Kruskal–Wallis pairwise comparison test, Benjamini–Hochberg corrected). *n*(wt − 4 dpe) = 29, *n*(wt − 1 dpe) = 19. Please note that the data presented in (*c*–*e*) is the same as in figure 3.

Mosquito flight behaviour in response to a synthetic human blend [11] (electronic supplementary material, table S1) and CO_2_ was recorded in a wind tunnel setup, as previously described [20]. The dimensions of the flight arena were 200 cm × 60 cm × 60 cm, illuminated by infrared light (850 nm) and visible white light of low intensity (less than 1 lux; figure 1*a*). Mosquito flight was captured at 60 frames s^−1^, and the filmed volume covered by the two cameras included the 120 cm at the upwind end of the flight arena (figure 1*a*, light grey box). The wind tunnel was adjusted to 27°C and 70% RH, and a wind speed of 0.22 m s^−1^. The odour stimuli were delivered at 0.4 l min^−1^, as previously described [20,21]. Briefly, metered CO_2_ of 1200 ppm (in 20.0% O_2_, 79.9% N_2_; Strandmöllen AB, Ljungby, Sweden) was presented using a glass hoop to create a turbulent plume [22]. The synthetic human blend was delivered by passing carbon-filtered and humidified air through a 100 ml Erlenmeyer flask containing a wick dispenser [23], with the blend at different concentrations diluted in pentane (≥95%, Carlo Erba Reagents, Emmendingen, Germany). This stimulus was presented using a glass tip, pointing upwind, to create a homogeneous plume. The dimensions and structure of the plumes were verified using smoke paper (Günther Schaidt SAFEX Chemie GmbH, Tangstedt, Germany).

#### (ii) Experimental procedure

Female mosquitoes were deprived of sucrose 17–18 h before the start of the experiment, but had *ad libitum* access to distilled water. One hour before the start of the experiment, mosquitoes were transferred to individual release cages using an aspirator, and kept in an incubator (27°C, 75 ± 10% RH) adjacent to the wind tunnel until use. For each trial, a release cage containing a single female was placed at the release point of the flight arena (figure 1*a*). After an acclimatization period of 2 min, the video recording was started, and the door of the release cage gently opened. If the mosquito did not enter the filmed volume within 5 min, the mosquito was scored as not responding. If the mosquito responded by entering the filmed volume, video capture was continued for up to 5 min, until the mosquito landed and remained resting for 10 s, or left the filmed volume for more than 1 min. Thirty females were tested for each genotype (or treatment), and each mosquito was only used once. For experiments including different genotypes (experiments II and III), the order of genotypes was randomized and time-balanced (i.e. a similar number of mosquitoes were used within each time block). All experiments were conducted in the scotophase, at Zeitgeber time 13.25–16.75 h (with Zeitgeber time 12 h referring to the start of the scotophase), the peak activity period of host-seeking *A. coluzzii*, as determined in a pilot experiment.

*Experiment I: dose–response.* To determine the optimal dose of the odour stimulus, a serial dilution of the synthetic human blend (electronic supplementary material, table S1) was prepared. The response rate of 30 non-blood-fed wild-type females (4 dpe) was recorded per dose.

*Experiment II: 1* *dpe*. Non-blood-fed teneral female mosquitoes of the five genotypes (wt, *OR39*^42/42^, *OR39*^42/+^, *OR39*^117/117^, *OR39*^117/+^) were tested at 1 dpe to the optimal dose of the synthetic human blend (*v*/*v* = 1/100; figure 1*b*). To compare to the flight behaviour of mature females, 4 dpe wild-type females were included as a control.

*Experiment III: 4* *dpe*. Non-blood-fed 4 dpe female mosquitoes of the five genotypes (wt, *OR39*^42/42^, *OR39*^42/+^, *OR39*^117/117^, *OR39*^117/+^) were tested at the optimal dose of the synthetic human blend established in experiment I (*v*/*v* = 1/100).

#### (iii) Data analysis

A mosquito was considered as responding to a presented odour stimulus if it entered the filmed volume within 5 min. The response rate per treatment was calculated as the number of mosquitoes responding divided by the total number of mosquitoes tested, as per common practice. A binomial generalized linear model followed by a *χ*^2^ test (R v.4.2.0 [24]) was used to test for the effect of the treatment (experiment I), genotype and age (experiment II) or genotype (experiment III). *Post hoc* pairwise comparisons were performed using the ‘emmeans’ package (R), corrected for multiple comparisons using the Tukey method. For analysis of the flight parameters in experiment II and III, the flight trajectories were reconstructed using EthoVision XT 14 and Track3D (Noldus Information Technology, Wageningen, The Netherlands), and consecutively analysed using customized Matlab (v.R2020a; MathWorks, Natick, USA) and R scripts (see also [20]). The following variables were calculated by Track3D and used for the analysis: position (*x*, *y*, *z*), angular velocity and tortuosity in the vertical plane (*x*–*y*) and in three dimensions. The output of the customized Matlab scripts included flight duration, proportion of flight spent ‘bouncing’ on the upwind screen (see figure 1*a*), proportion of flight spent in the volume of interest (VOI) (figure 1*a*), saccade frequency and turn consistency (i.e. the mean tortuosity after a saccade). For most of the analysis, the data points, in which the position of the mosquito was less than 6 cm away from the upwind screen were excluded, since the interaction with the physical boundary (bouncing) likely affected mosquito flight. The flight duration was calculated as the time spent in the filmed volume, excluding the ‘bouncing’, whereas the proportion of flight spent ‘bouncing’ was the number of frames closer than 6 cm to the upwind screen, divided by the total number of frames. To approximate the volume in which mosquitoes were likely to encounter odour filaments of a given odour stimulus, the plumes were initially visualized using smoke paper (see above), and the VOI defined as a cylinder in space (diameter 14 cm), centred within the flight arena. The proportion of flight spent in the VOI was calculated as the number of frames with a position within the VOI divided by the total number of frames. For analysing flight saccades, positions closer than 6 cm to any wall and 20 cm to the upwind screen were excluded, and trajectories shorter than 30 frames (0.5 s) removed. The remaining flight trajectories were smoothed by a moving average over a window of six frames (0.1 s). The centre of a saccade was defined as the local maximum of a bout characterized by an angular velocity of more than 2000 deg s^−1^. The saccade frequency is the number of saccades per second. The turn consistency is defined as the mean tortuosity in the 60 frames (1 s) after a saccade, and thereby a measure for the likelihood of the mosquito to continue in a saccadic flight pattern. Tortuosity is calculated as the flight path between two points in three-dimensional space, divided by the length of the straight line connecting these two points, with a value of 1 indicating a straight flight path, and values greater than 1 × *x* denoting a flight path *x* times longer than the straight line. Furthermore, a principal component analysis (PCA) was employed to visually demonstrate variance between the groups. The PCA was optimized for maximal differences between teneral (1 dpe) and mature (4 dpe) wild-type mosquitoes, and the final model included the five variables listed above. For each of the variables, a Dunn Kruskal–Wallis multiple comparison *post hoc* test with Benjamini–Hochberg correction was used for pairwise comparison between the groups (R package ‘FSA’ v. 0.9.1).

### (d) Single sensillum recordings

*In vivo* SSRs were used to identify and functionally characterize the OSN expressing *OR39* in wild-type, homozygous and heterozygous females, and were carried out as previously described [11,25]. Non-blood-fed females (4–10 dpe) were cold-anesthetized and mounted on a double-sided tape attached to a microscope slide. Another piece of tape covered the remaining body, with the exception of the head. A tungsten reference electrode was inserted into the eye of the mosquito, and a tungsten recording electrode was inserted into the base of a trichoid sensillum, type E2 (TE2), previously identified to respond to sulcatone and 1-hexanol (electronic supplementary material, figure S1). Serial dilutions of neat compounds (sulcatone, ≥98%, Sigma-Aldrich, Merck, Darmstadt, Germany; nonanal, 95%, Acros Organics, Thermo Fisher Scientific; eugenol, 99%, Sigma-Aldrich; phenol, ≥99%, Sigma-Aldrich; the latter three VOCs were included to differentiate between TE sensilla with similar response spectra) were prepared in pentane or water, and 15 µl of each aliquot was used for the preparation of an odour delivery pipette. The stimulus was presented to the antennae of the mosquito by a stimulus controller (Ockenfels Syntech, Buchenbach, Germany). The TE2 sensilla in homozygous mutants were identified based on the characteristic response of the A neuron to eugenol and phenol. Spike rates of individual OSNs were calculated as the spikes recorded during the 0.5 s stimulation period minus the number of spontaneous spikes during the preceding 0.5 s, and then multiplied by two. A linear mixed-effects model (R package ‘lme4’ v. 1.1–27.1), with random intercept for sensillum ID to control for repeated measures, was fit to the spike rate, and the effect of genotype and dose were tested using an ANOVA. The ‘emmeans’ R package (v.1.7.3) was used for pairwise comparisons between the lines and corrected for multiple comparisons using the Tukey method.

## 3. Results

### (a) Mosquito flight behaviour is modulated by adult maturation

Mosquito flight behaviour was observed in a wind tunnel setup (figure 1*a*). In the first experiment, using mature wild-type females, the optimal dose of the synthetic human blend, in combination with CO_2_, was established (1/100; pairwise comparison to the solvent, *p* = 0.0004; figure 1*b*), and used for further experiments. While only 26.7% of mature females entered the filmed volume when presented with the solvent in a background of 1200 ppm CO_2_, a 1/100 dilution of the synthetic human blend elicited flight in 83.3% of mosquitoes. The second experiment allowed for testing the effect of age on mosquito response rate and flight behaviour, by comparing teneral and mature wild-type mosquitoes. Mosquito age had a significant effect on the response rate (*χ*^2^ test, *p* < 0.001), although the pairwise comparison between the two age groups missed significance (*p* = 0.053; figure 1*c*). When analysing the flight parameters of responding mosquitoes, the flight duration was significantly different between the two age groups (Dunn Kruskal–Wallis test, *p* = 0.045; figure 1*d*), with mature females on average being airborne 2.7 times longer than teneral females. Moreover, the flight of mature females was characterized by a significantly longer time ‘bouncing’ at the upwind screen (*p* = 0.00042; figure 1*e*), a higher saccade frequency (*p* = 0.0499) and turn consistency (*p* = 0.00053; figure 1*f*).

### (b) Mutagenesis of *OR39* results in a non-functional OSN

**Figure 2.**
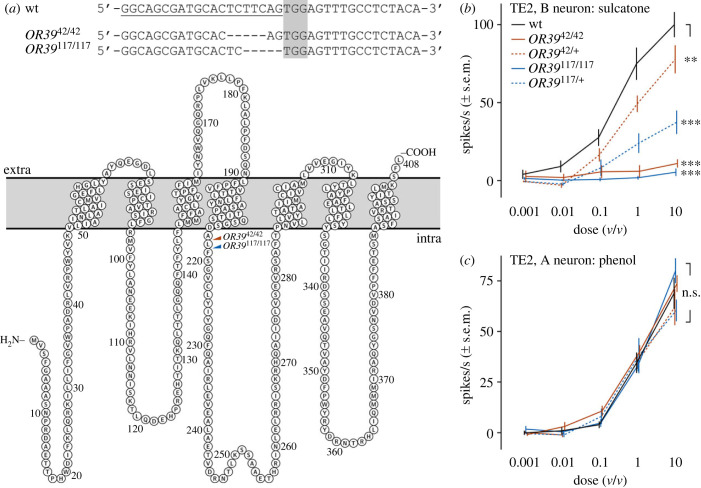
Mutagenesis and function of *Anopheles coluzzii* OR39. (*a*) CRISPR–Cas9 mutagenesis of *OR39*. (*a*) Alignment of the targeted nucleotide sequence of *OR39* in wild-type (wt) and mutant (*OR39*^42/42^, *OR39*^177/117^) mosquitoes. The protospacer is underlined in the wt and the protospacer adjacent motif (PAM) indicated by a grey box. In both lines, the mutation consists of a 5 bp deletion, encompassing the expected mutagenesis site 3 bp upstream of the PAM. (*b*) Protein snake plot of the wild-type OR39 protein with the target site for both mutations highlighted. Transmembrane domains were predicted using trRosetta [26] and RSCB PDB pairwise alignment viewer [27], and the snake plot was made using the open-source protein feature visualization tool PROTTER [28]. (*b*) Response of the TE2-B neuron to increasing doses of sulcatone and (*c*) the TE2-A neuron to increasing doses of phenol in mature wild-type, heterozygous and homozygous mutant females. Pairwise comparison to the wild-type using ‘emmeans’ (R). *n*(wt) = 12–15, *n*(*OR39*^42/42^) = 14, *n*(*OR39*^42/+^) = 8, *n*(*OR39*^117/117^) = 4–5, *n*(*OR39*^117/+^) = 10, *p* < 0.05.

Two knockout-lines targeting the *A. coluzzii OR39* gene were generated using CRISPR–Cas9 genome editing. The two independent lines, *OR39*^42/42^ and *OR39*^117/117^, each carried distinct deletions of 5 bp after position 652 and 653, respectively (figure 2*a*), resulting in a frame-shift and thus a premature stop codon. The protein sequences were predicted to be diverging from the wild-type after position 217 and 218, respectively, both truncated after 252 amino acids. To assess the function of OR39 in wild-type, heterozygous and homozygous mutant mosquitoes, SSRs were conducted from TE2 sensilla, in which the B neuron was demonstrated to respond to the key ligand of OR39, sulcatone (electronic supplementary material, figure S1). The sensitivity of the TE2-B neuron to sulcatone was abolished in both homozygous mutant lines (pairwise comparison to the wild-type, *p* < 0.0001; figure 2*b*) and significantly reduced in the heterozygous insects (*p* < 0.01). The function of the A neuron was not affected by the knockout (*p* = 0.64; figure 2*c*). Besides the TE2 sensilla, at least two other trichoid sensilla contained OSNs responding to sulcatone (electronic supplementary material, figure S1).

### (c) Knockout of *OR39* does not affect the onset of host seeking in teneral females

**Figure 3.**
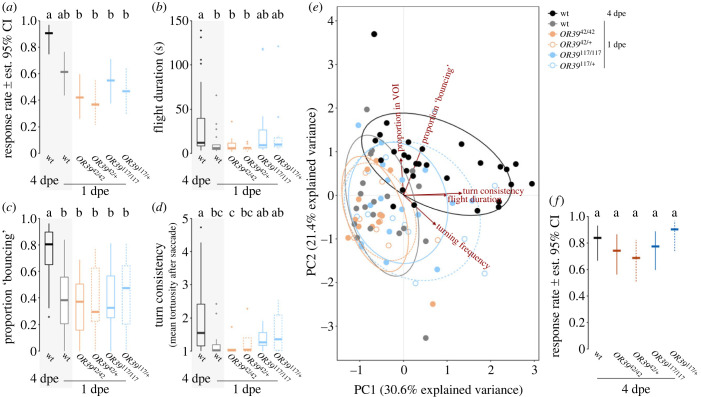
Mosquito flight in OR39 knockout mosquitoes. (*a*) Response rate of mature (4 dpe) wild-type females, as well as teneral (1 dpe) wild-type and mutant females to the synthetic human blend and CO_2_ (experiment II). Different letters denote significant differences between the genotypes (pairwise comparison using ‘emmeans,’ corrected with the Tukey method). *n*(per treatment) = 30–32, *p* < 0.05. (*b*) Flight duration, (*c*) proportion of flight spent ‘bouncing’ at the upwind screen and (*d*) turn consistency (i.e. the mean tortuosity after a saccade). Please note that the data for the wild-type mosquitoes (grey boxes) is also shown in figure 1. Boxes represent upper and lower quartiles, whiskers denote 1.5 times interquartile distance, crosses outliers and thick horizontal lines the median. Different letters denote significant differences between the treatments (Dunn Kruskal–Wallis pairwise comparison test, Benjamini–Hochberg corrected). *n*(wt − 4 dpe) = 29, *n*(wt − 1 dpe) = 19, *n*(*OR39*^42/42^ − 1 dpe) = 12, *n*(*OR39*^42/+^ − 1 dpe) = 9, *n*(*OR39*^117/117^ − 1 dpe) = 17, *n*(*OR39*^117/+^ − 1 dpe) = 13. (*e*) Principal component analysis (PCA) of select flight parameters: proportion of flight within the VOI, proportion of flight ‘bouncing’ at the upwind screen, turn consistency, flight duration and saccade frequency. The PCA was optimized for maximal separation of the two wild-type groups. *n* = 9–29. (*f*) Response rate of mature wild-type and transgenic mosquitoes to the synthetic human blend and CO_2_ (experiment III). *n*(per genotype) = 31–32.

When comparing the response rate of mutant teneral females and the wild-type controls to the optimal dose of the synthetic human blend and CO_2_ (experiment II), the age (*χ*^2^ test, *p* < 0.001), but not the genotype (*p* = 0.26) of the mosquitoes contributed significantly to the observed responsiveness. The mature wild-type mosquitoes displayed a significantly higher response rate than teneral mutant females (emmeans, *p* < 0.05), but there was no difference in response rate among the genotypes of teneral females (*p* > 0.40; figure 3*a*). In addition, the detailed analysis of the flight behaviour of those mosquitoes that responded, showed no difference among the teneral females for the flight duration (Dunn Kruskal–Wallis; *p* > 0.12; figure 3*b*), proportion of flight spend ‘bouncing’ (*p* > 0.96; figure 3*c*) and saccade frequency (*p* > 0.19). The turn consistency was not significantly different between teneral wild-type and mutant mosquitoes (*p* > 0.06; figure 3*d*). When comparing all analysed flight parameters, using PCA, mosquitoes generally clustered by age, but not genotype (figure 3*e*). There was no difference between the mutant lines and the wild-type mosquitoes for either the response rate (emmeans, *p* > 0.64; figure 3*f*) or the flight parameters (Dunn Kruskal–Wallis; *p* > 0.13; electronic supplementary material, figure S2) when testing mature females (experiment III), indicating the absence of off-target effects of the CRISPR–Cas9 mutagenesis.

## 4. Discussion

Female mosquitoes demonstrate an age-dependent behavioural shift in their response to human odour, which correlates with changes in the transcript abundance of chemosensory genes and the sensitivity of the peripheral olfactory system [5,8–12,14]. Previous research identified *AcolOR39*, encoding a receptor tuned to one of the major compounds of human odour, sulcatone [11,21], as a candidate gene regulating the acquisition of host seeking in the malaria mosquito, *A. coluzzii* [11]. CRISPR–Cas9 knockout of *OR39* abolished the physiological response to sulcatone. While high-resolution behavioural analyses replicated the reported age-dependent change in response to human odour [5,8–12], knockout of *OR39* affected neither the response rate nor the flight behaviour of teneral and mature mosquitoes. Available data thus suggests that OR39 alone may not play a critical role in regulating host seeking, and that sulcatone is detected by redundant pathways in the olfactory system, which regulates the onset of host seeking in *A. coluzzii*.

While sulcatone is not unique to human odour [21,23,29–31], it is a breakdown product of human sebum [32], and occurs in exceptionally high proportion in human skin emanations [21,30]. Tested alone, sulcatone may elicit both aversion and repellence [33–35]. Sulcatone is thus a compelling VOC mediating the age-dependent behavioural inhibition to human odour [11]. Although OR39 is the only OR tuned to sulcatone that significantly changes in abundance in an age-dependent manner in *A. coluzzii* [11], several other receptors detect sulcatone at physiologically relevant [11] and elevated concentrations [15], as confirmed through *in vivo* screening of all functional types of trichoid sensilla in this species. While sulcatone regulates aversion in 1 dpe *A. coluzzii* [11], knockout of *OR39* does not provide a behavioural phenotype and indicates a complex regulation of the acquisition of host seeking through combinatorial coding in the olfactory pathway.

Besides OR39, the study by Omondi *et al.* [11] highlighted three other ORs tuned to salient human-derived VOCs that change significantly in transcript abundance (upregulated) during adult maturation. While OR1 and OR2 are mostly responsive to phenolic compounds and benzaldehyde, OR75 is tuned to a group of monoterpenoids, of which limonene is the most abundant compound in human odour [11]. A similar gene editing approach as the one used here may be of interest to assess the involvement of these candidate ORs in regulating the acquisition of host seeking. Another pathway, with a potentially large impact on the onset of host seeking, is the CO_2_-chemosensory system, which detects the general presence of a breathing host [36–39]. As this pathway exhibits similar age-dependent regulation, in terms of OSN sensitivity and gene expression [14], it would be a compelling experiment to increase expression of *GR22*, encoding for one of the subsets of the CO_2_ receptor, in teneral females, to investigate its contribution to the onset of host seeking. Taken together, the acquisition of host seeking in mosquitoes is likely regulated by not one, but a set of genes, including those directly related to the detection of human odour.

Anthropophilic mosquitoes are an interesting model with which to study shifts in odour valence, since there is a strong selection pressure for females to react adequately and context-dependently to human host odour [1,2,4]. Human odour gains and loses its attractive properties several times during the lifespan of a female mosquito, regulated not only by age, but also by mating status, nutritional state, gonotrophic cycle and circadian rhythmicity (reviewed by Klowden [6] and Hill & Ignell [7]). The often rapid modulation of the response to human host odour is achieved by a highly plastic peripheral olfactory system, with context-dependent expression of chemosensory and neuromodulatory genes [11,12,40–42]. As a result, shifts in odour valence may be a consequence of combinatorial coding, in which the various input channels may be differentially regulated resulting in the observed range of responses to human odour.

## 5. Conclusion

This study, although concluding that *OR39* alone does not modulate the behavioural transition towards host-seeking competence in *A. coluzzii* females, made use of the recent advancement of genetic tools in mosquitoes and raised the bar by targeting one of the candidate genes regulating the acquisition of host seeking, thereby allowing for testing for causality. Future studies will need to address the role of OR39 in *A. gambiae s. l.* chemical ecology, and continue to investigate the causative molecular mechanism of the acquisition of host-seeking behaviour in mosquitoes.

## Data Availability

Behavioural and physiological data: https://doi.org/10.5061/dryad.h70rxwdpz [43]. Supplementary material is available online [44].
